# Turn-taking fluency in free conversations with individuals diagnosed with schizophrenia

**DOI:** 10.1038/s41537-025-00678-y

**Published:** 2025-11-04

**Authors:** Tifenn Fauviaux, Ghilès Mostafaoui, Richard C. Schmidt, Mathilde Parisi, Victor Vattier, Dorra Mrabet, Delphine Capdevielle, Stéphane Raffard, Ludovic Marin

**Affiliations:** 1 EuroMov Digital Health in Motion, Univ Montpellier, IMT Mines Ales, Montpellier, France; 2 CY Cergy Paris Université - ETIS UMR 8051, Cergy-Pontoise, France; 3 College of the Holy Cross, Worcester, MA USA; 4 Université Paul Valéry Montpellier 3, EPSYLON EA, Montpellier, France; 5 University Department of Adult Psychiatry, CHU Montpellier, Montpellier, France

**Keywords:** Diseases, Schizophrenia

## Abstract

Disruptions in language processing observed in Individuals diagnosed with schizophrenia (ISZ) are likely to impair turn-taking fluency and social functioning. While turn-taking research in ISZ is limited and mostly interview-based, this study examines fluency differences between ISZ and controls in free conversations and their links to social outcomes and symptoms. We recruited 20 ISZ, 20 healthy interacting partners (IP), and 20 matched controls (MAT). Each IP, unaware of the ISZ diagnosis, had a 6-min conversation with an ISZ and a MAT, and then rated their willingness to interact again. Voice recordings were analyzed for pauses, gaps, and overlaps. Results revealed that conversations with ISZ featured fewer overlaps, more and longer gaps, and extended pauses. Additionally, the gap duration influenced participants’ willingness to engage in future interactions. ISZ symptoms disrupted their speech and were linked to longer gaps and pauses in their partner’s speech. This study extends fluency research in ISZ by shedding light on natural conversational dynamics.

## Introduction

Individuals diagnosed with schizophrenia (ISZ) often experience significant impairments in social functioning, the ability to engage in and sustain social interactions^1^. These difficulties stem from core symptoms of the disorder, including positive symptoms (e.g., delusions, hallucinations), negative symptoms (e.g., reduced motivation, diminished expression), and formal thought disorder^2^. The latter involves poverty of speech and disorganized discourse leading to communication difficulties^3,4^. Previous studies found that ISZ experience difficulties across various language production and perception levels. Production difficulties span both higher-level cognitive processes related to semantics and syntactic processes, as well as more fundamental aspects of speech like acoustic production (i.e., slower speech rates, longer pauses, and lower speaking proportion)^5–8^. Concerning speech perception deficits, studies have found impairment in central auditory processing, along with reduced social cognitive abilities such as the perception, interpretation, and processing of social information^9–12^. Such perception and production language deficits could make it challenging for ISZ to follow the implicit conversation rules^7^.

Conversation is a dynamic process wherein individuals alternate between speaking and listening, forming the basis of turn-taking^13^. In this universal social system, speakers tend to minimize overlap (when a listener speaks before the speaker has finished their turn) and long gaps (the silence between different speakers’ turns)^14,15^. The result is a coordinated sequence of turns with inter-turn intervals typically around 200 ms^14,16^. Since spoken word conceptualization and production normally takes around 600 ms, the listener must anticipate when the current speaker will conclude their turn^17–19^. Consequently, managing the precise timing of a conversation requires cognitive processes. The listener must understand the current turn and anticipate the next one, formulate a response, and deliver it at the expected endpoint^18–20^. The brief gaps between turns, used as a proxy for speech latency, reflect the respondent’s temporal behavior and indicate how efficiently interlocutors manage dialog coordination^21,22^. Maintaining well-timed latencies supports smooth transitions and overall dialog fluency.

Fluency includes the speaker’s ability to produce flowing speech with a high production rate, without pauses, hesitations, fillers, and corrections^23,24^. Along this line, studies on perceived fluency in nonnative speech have found that two of the strongest predictors are the mean number and duration of silent pauses, where ‘silent pause’ refers specifically to the silence between two continuous stretches of speech produced by the same speaker^25–27^. On the dyadic level, fluency lies in the capacity to maintain flow from side to side of turn boundaries - including short gaps and overlaps- and is a key indicator of social outcomes, mirroring both the context and quality of conversation^24,28,29^. For example, turn-taking can vary with the number of participants and whether the setting is informal or institutional (i.e., with a doctor or researcher), where roles can be more asymmetrical^14,30^. Moreover, research on neurotypical participants shows that fast responses foster connection^29^, while long gaps are linked to negative outcomes like less compliance, weaker affiliation, and lower shared cognition^28,31–33^.

Because turn-taking mediates fluency and social connection, disruptions in language processing, such as those seen in ISZ, are likely to impair the smooth coordination of conversational turns and social functioning^5^. However, despite the relevance of speech timing as a marker of communicative difficulties, research on turn-taking in ISZ remains limited and inconsistent. A study analyzed speech from psychiatric interviews and found that ISZ speech was more fragmented, presenting abnormally long gaps caused by turn-taking delay^34^. In triadic moral dilemma discussions, another study showed that ISZ presence altered interlocutors’ behavior, leading to longer gaps and more pauses, even when the diagnosis was undisclosed^35,36^. Finally, another study found more overlaps and mutual silences in ISZ interactions during semi-structured interviews, though gap rates did not differ. Moreover, psychiatrists participated more with ISZs compared to dialog with controls, and negative symptoms were linked to turn-taking patterns, especially mutual silence^37^.

These findings confirm disfluencies in ISZ interactions, though the unnatural and institutional context may have influenced them. While triadic conversation requires more cognitive effort, psychiatrist interviews impose a more structured or oriented interaction. To date, no study has specifically examined the turn-taking dynamics of free conversations between ISZ and control participants, leaving a significant gap in our understanding of how these interactions unfold in real-life contexts. Moreover, given ISZ’s social functioning deficits and the link between fluency and social bonding among neurotypical individuals, exploring how turn-taking fluency affects social outcomes in clinical populations would be valuable. Importantly, because the timing of turn-taking emerges as a co-constructed process between two interacting partners, one must consider both the dyadic outcome and the individual contributions that shape it.

This study aims to investigate turn-taking fluency in free conversations between ISZ and controls. We recruited 20 ISZ, 20 interacting partners (IP), and 20 matched participants (MAT). Each IP, unaware of the ISZ diagnosis, engaged in a 6-min free conversation once with an ISZ and once with a MAT. At the end of the interaction, participants answered questions about their willingness to engage in future interactions as a social outcome. We recorded their voices and extracted metrics (gaps, overlaps, and pauses) used to assess individual and dyadic fluency. These metrics were analyzed at both the dyad level to assess overall fluency and the individual level to understand each participant’s contribution, considering the influence of individual differences on dyadic interactions. Moreover, we seek to explore the relationship between turn-taking fluency and willingness to engage in future interactions as our social outcome. Finally, we explored whether disfluencies found in ISZ could be related to psychopathological dimensions^37,38^. Based on the previous findings of the literature:

(H1) We expect to find a decrease in dialog fluency in ISZ interactions driven by overlaps, gaps, and pauses:

- **Overlaps** We expect a higher number of overlaps in ISZ interactions^37^ and will further examine their duration, along with how each participant contributes to them.
- **Gaps** We expect no significant differences in the proportion of gaps between ISZ and control interactions^37^ but hypothesize longer gap durations in ISZ interactions^34^, with both ISZ and control contributing to this increase^34,36^.
- **Pause** We expect ISZ conversations to have more and longer pauses, with ISZ participants and their partners contributing to the increased duration^7,35^.

(H2) As speech fluency decreases in ISZ interactions, we expect this to negatively affect the willingness of participants to continue the conversation.

(H3) We anticipate that the reduced fluency in ISZ interactions will correlate with certain psychopathological dimensions of schizophrenia, particularly negative symptoms^37–40^.

## Materials & methods

### Study design

The current study is part of the Enhancer project (ANR-22-CE17-0036), which investigates the dynamics of speech and gesture in social interactions involving individuals diagnosed with schizophrenia. The French Ethical Committee ethically approved this study - Comité de la Protection des Personnes (2024-A00553-44) - along with a compensation of 50 euros for the healthy participants. After receiving a written information letter, all participants provided informed consent before the experiment.

This study employed a specific design where healthy IPs interacted with two distinct partners separately: an ISZ and a healthy MAT. It resulted in triplets, each consisting of one IP interacting once with ISZ (IPs_ISZ) and once with MAT (IP_MAT) (for better clarity, we will refer to “IPs” to name the interacting partner of ISZ). ISZ and MAT were matched on age (U = 160.5, *p* = 0.29) and sex, allowing only comparison based on diagnostic status^41^.

### Participants

We recruited 60 participants (20 ISZ, 20 MAT, and 20 IP) for 20 triads to meet the required sample size, 20 ISZ (age: M = 32.3, SD = 9.4, 6 women, 14 men), 20 MAT (age: M = 31.1, SD = 11.9, 6 women, 14 men), and 20 IP (age: M = 26.3, SD = 6.5, 6 women, 14 men). All MAT and IP participants were recruited in Montpellier (France), presented no history of psychosis or neurological or psychiatric disorders, and were not taking any medication known to impact cognition. ISZ participants were recruited from the University Department of Adult Psychiatry in Montpellier. All participants spoke French. Inclusion criteria required a DSM-5 diagnosis of schizophrenia, clinical stability, and ongoing antipsychotic treatment at the time of participation. Symptomatology was assessed using the Positive and Negative Syndrome Scale (PANSS)^42^, and medication doses were converted to chlorpromazine (CPZ) equivalents to allow comparison across different antipsychotic treatments. Moreover, we used the Montreal Cognitive Assessment (MOCA) to control for cognitive differences^43^, the Beck Depression Inventory II (BDI-II) for depression^44^, the French National Adult Reading Test for premorbid intelligence (fANRT)^45^, and the Positive and Negative Affect Schedule (PANAS) for emotions^46^ (Table 1). No differences were found for these measures (*p* > 0.05), except for MoCA (*p* = 0.02).

**Table 1:** Participants descriptive.

	IP (n = 20)		MAT (n = 20)		ISZ (n = 20)				
Information	*M*	*SD*	*M*	*SD*	*M*	*SD*	Statistics	*p*-value	
							*(MAT vs ISZ)*		
Age	26.3	6.5	31.1	11.9	32.3	9.4	*U* = 160.5		*p* = 0.29
fNART	29.5	3.9	28.6	5.3	26.8	7.4	*U* = 211.5		*p* = 0.37
MoCA	28.7	1.1	28.7	1.8	26.7	3.3	*U* = 256.5		*p* = 0.02
PANAS positive	38.7	5.1	35.1	4.0	32.9	7.0	*t* = 1.17		*p* = 0.25
PANAS negative	19.5	4.3	19.7	6.7	19.2	5.3	*t* = 0.30		*p* = 0.77
BDI-II	9.0	5.1	9.0	9.4	11.6	9.0	*U* = 140.5		*p* = 0.25
PANSS positive					12.7	5.4			
PANSS negative					19.0	11.2			
PANSS general					36.5	17.4			
CPZ equivalent dose in mg					302.4	263.2			
Illness duration					9.9	10.0			
Gender (M/W)	14/6		14/6		14/6				

*IP* interacting partner, *MAT* healthy subjects matched in age and sex, *ISZ* individuals with a diagnosis of schizophrenia, *CPZ* Chlorpromazine.

### Task

Each dyad participated in a 6-min free conversation. To avoid long silences while deciding on a topic, we provided each participant with a document containing various themes, such as movies, holidays, etc. Participants were instructed to select two topics to discuss, but could switch anytime. The instructor left the room to encourage spontaneous conversation. Participants were seated approximately 1.5 meters away and wore a lapel microphone (Hollyland LARK 150) to record their speech. The first four triads wore lapel microphones, but due to poor sound quality and diarization issues, the remaining triads used headsets for clearer audio. Despite increased volume, crosstalk persisted. Microphones were connected to a Zoom H6 recorder, and speech was recorded at 44,000 Hz and saved as .wav files. The lip-to-microphone distance and angle were not standardized during recordings, but were adjusted to ensure the comfort of each participant.

### Procedure

Upon arrival, participants (ISZ or MAT) met their partner in a room. IP interacted twice, once with the ISZ and once with the MAT, with the interaction order counterbalanced. Before the experiment, participants were given an information letter explaining the study; they subsequently provided their informed consent and completed the First Impression Scale^47^, BDI, and PANAS. Before beginning the main task, participants took part in a four-minute icebreaker conversation intended to help them feel more comfortable interacting with each other^48^. They were then invited to engage in the free conversation task described above. After the interaction, participants completed the Willingness to Interact Scale^49^, a five-point Likert scale assessing willingness to engage in future social situations. Originally consisting of 6 items (e.g., “take advice,” “take the bus”), we added 3 items (e.g., “get to know better,” “liking,” “perceived similarity”). These items were adapted from previous research on interpersonal attraction and social evaluation^41,50–52^. Final items used the same Likert scale, and demonstrated excellent internal consistency (α = 0.92; 95% CI: [0.89, 0.94]).

### Extraction of turn-taking metrics

Each .wav file was processed in WavePad Audio Editor to reduce room reverberation and amplify the voice when the microphone was distant. The files were then exported to ELAN, where an automatic Voice Activity Detection using Silence Recognizer was applied to identify and mark silent segments. The minimal silence duration was chosen to be 200 ms^27^. Due to crosstalk in our audio file, we manually corrected speech segments to assign them to the correct speaker. Audible in-breaths were removed, and filled pauses and laughter were classified as speech activity^30,53^. The resulting speech segments are defined as Inter-Pausal Units (IPUs), maximal sequences of words surrounded by any silence exceeding 200 ms^27^. For each IPU, we manually determined whether it was a backchannel (BC), namely, a short expression (i.e., “ok”) used by the listener to express attention or interest^54,55^.

**Fig. 1:**
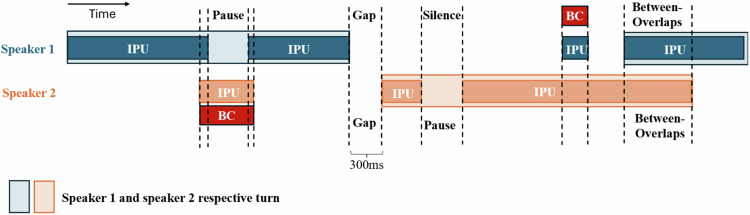
Illustration of key turn-taking metrics between two speakers (adapted from ref. ^56^). The metrics represented correspond to Inter-Pausal Unit (IPU), Backchannel (BC), Pause, Gap, and Between-overlaps (simply referring to overlaps). The metrics are represented at the individual level (reflecting the specific involvement of each participant) and at the dyadic level (representing the interaction between the two participants). For example, when speaker 1 finishes his turn, speaker 2 takes 300 ms to respond. This gap is recorded for the dyad and is also attributed to Participant 2, as he initiated the delay.

The binary speech files were processed in MATLAB R2021B to derive turn-taking metrics based on methods from Heldner and Edlund and Levinson and Torreira, incorporating backchannels^19,56^. The algorithm identifies (1) a turn as a sequence of uninterrupted IPUs from one speaker (except for BCs that do not constitute a claim for a turn)^27,55^; (2) Gaps as periods of silence between speakers; (3) Overlaps when one speaker starts before the other finishes; (4) Pauses as silence within a speaker’s turn (Fig. 1).

### Variables of interest

Gaps, overlaps, and pauses were extracted as our interest variables. For each variable, we extracted both the structural properties (the total number relative to the total number of possible turn transitions, expressed in %) and temporal properties (the median duration expressed in ms, and the total duration relative to the total speaking duration, expressed in %). All metrics were extracted both at the dyadic level and the individual level (Fig. 1). Data from binary speech files and the analytic code of statistical analyses can be found on the Open Science Framework (https://osf.io/g97hn/).

### Statistical analysis

We first tested normality with the Shapiro–Wilk test and variance with Levene’s test. Only Shapiro–Wilk indicated non-normality in some variables. We applied natural logarithmic transformation to all our non-normally distributed variables, as is commonly done in psychosocial research^57–59^.

To test our first hypothesis, we fitted linear mixed-effects models (LMMs) using the *lme4* package in R^60^, with triad as a random intercept. We ran two separate sets of models: one testing the effect of the dyad (IP_MAT vs. IPs_ISZ; reference = IP_MAT), and another testing participant role (IP vs. IPs and MAT vs. ISZ; reference = IP), to assess whether IPs adapted their behavior depending on their partner:

*lmer (dependent_variable ~ dyad or participant* + *(1|triad))*

To test whether dyad or participant factors improved model fit, each model was compared to a null model (random intercept only) using AIC, BIC, and likelihood ratio tests. We also assessed whether adding order (i.e., which dyad occurred first) and its interaction with dyad or participant further improved fit. The dyad factor improved model fit for all variables except Overlaps median duration, Overlaps total duration, and Pause total number. A dyad × order interaction best explained the Gaps’ total and median duration. In participant models, the participant factor improved fit for all variables except for Overlaps median duration, Overlaps total duration, and Gaps median duration. For Gaps total duration, a participant × order interaction improved fit, and for Pause median duration, additive effects of dyad and order provided the best fit (see Supplementary Tables 1–2 for model comparison details). Analyses were restricted to these validated models. P-values were computed using the *lmerTest* package^61^. When multiple tests were run on the same data, we corrected for multiple comparisons using the False Discovery Rate (FDR)^62^.

## Results

### Overlaps

#### Dyad level

**Fig. 2:**
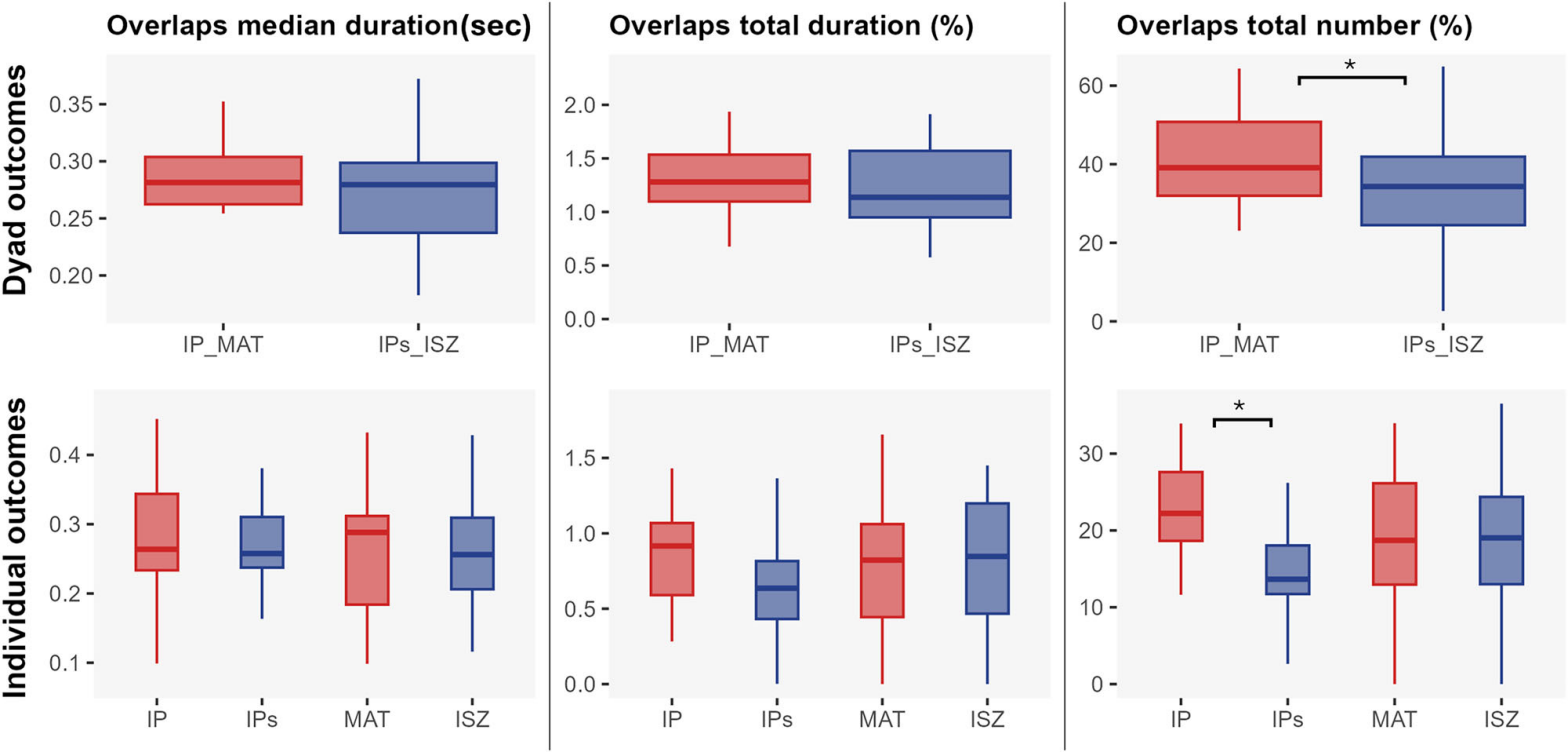
Boxplots of overlap metrics at dyadic and individual levels. The dyadic level is illustrated on the top row and the individual level on the bottom row. The first column displays the median duration of overlaps (in seconds), the second column shows the total overlap duration (in %), and the third column represents the total number of overlaps (in %). Red boxplots correspond to dyads where the interacting participant was IP and MAT (IP_MAT), while blue boxplots correspond to dyads involving IPs and ISZ (IPs_ISZ). Individual-level comparisons are made between IP and IPs and between MAT and ISZ. * denotes *p* < 0.05, ** *p* < 0.01, and *** *p* < 0.001.

The Linear Mixed Model results indicated a significant dyad effect on the total number of overlaps (*IP_MAT vs. IP_ISZ*: *b* = −8.34, *p* = 0.049, *d* = 0.71, 95% CI [0.03, 1.39]), with significantly more overlaps occurring in the IP_MAT (M = 41.9%) dyad compared to IPs_ISZ (M = 33.5%) (Fig. 2).

#### Individual level

The Linear Mixed Model results indicated a significant effect of the participant on the total number of overlaps for IP vs. IPs (*IP vs. IPs*: *b* = 8.27, *p* = .018, *d* = 1.11, 95% CI [0.45, 1.76]), with significantly more overlaps produced by the IP interacting with the MAT (M = 22.6%) compared to the IPs interacting with the ISZ (M = 14.3%) (Fig. 2).

### Gaps

#### Dyad level

**Fig. 3:**
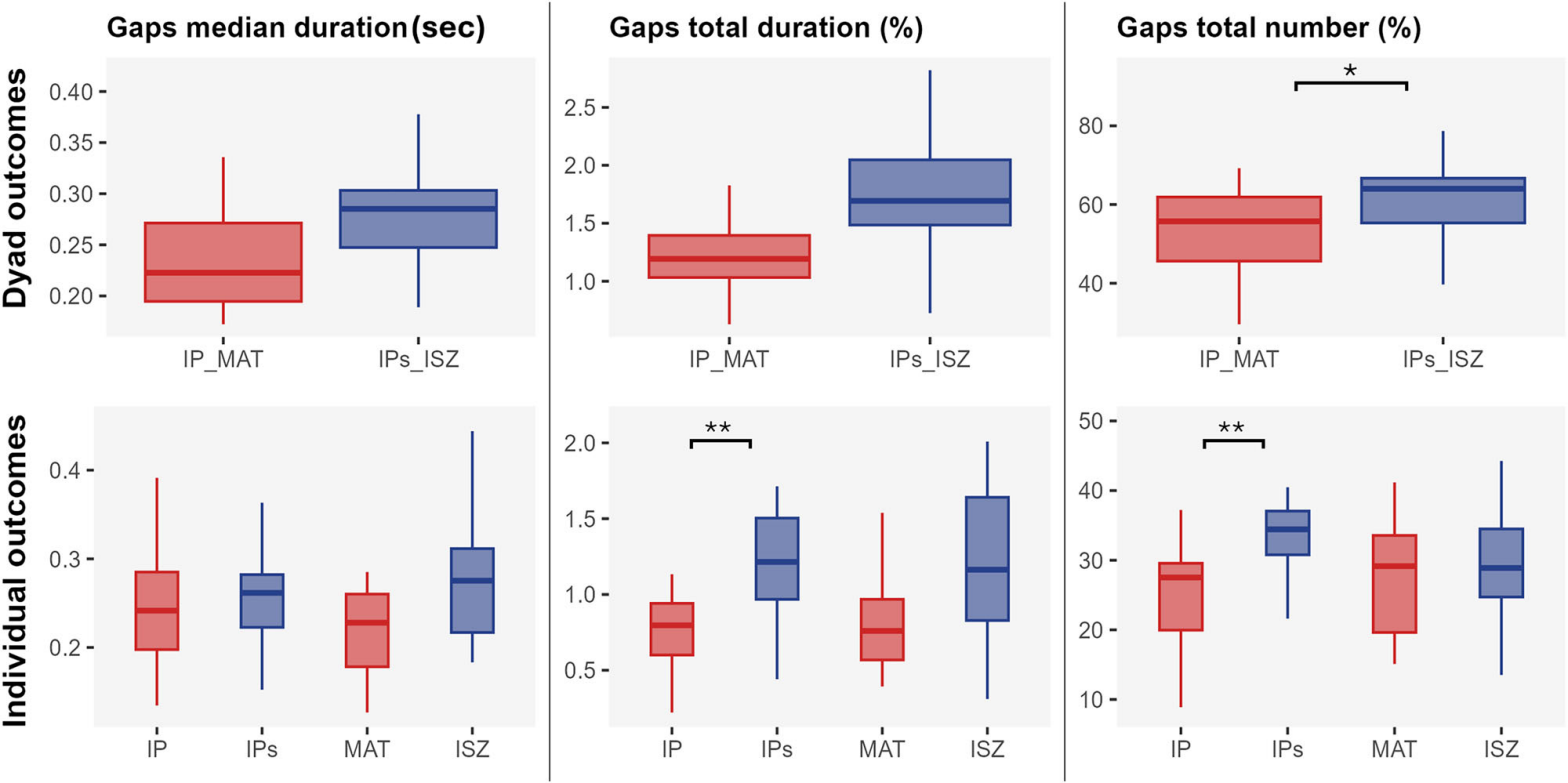
Boxplots of gap metrics at dyadic and individual levels. The dyadic level is illustrated on the top row and the individual level on the bottom row. The first column displays the median duration of gaps (in seconds), the second column shows the total gap duration (in %), and the third column represents the total number of gaps (in %). Red boxplots correspond to dyads where the interacting participant was IP and MAT (IP_MAT), while blue boxplots correspond to dyads involving IPs and ISZ (IPs_ISZ). Individual-level comparisons are made between IP and IPs and between MAT and ISZ. * denotes *p* < 0.05, ** *p* < 0.01, and *** *p* < 0.001. Order effects reported in the text are not displayed in the figure.

The Linear Mixed Model for gaps total number indicated a significant effect of the dyad (*IP_MAT vs IPs_ISZ*: *b* = 10.26, *p* = .043, *d* = −0.89, 95% CI [−1.59, −0.20]). Significant dyad × order interactions were found for gap total and median durations. In the IPs_ISZ dyad, gaps were longer when the conversation started with ISZ versus MAT (total: *p* = .049, *d* = 1.32, 95% CI [0.07, 2.57]; median: *p* = .043, *d* = 1.34, 95% CI [0.31, 2.36]). No order effect appeared in the IP_MAT dyad. Overall, the IP_MAT dyads produced shorter median gaps (M = 0.24 ms, M = 0.30 ms), shorter total gap duration (M = 1.26%, M = 1.74%), and fewer gaps (M = 52.56%, M = 62.82%) compared to the IPs_ISZ dyads (Fig. 3).

#### Individual level

The Linear Mixed Model indicated a significant effect between IP vs IPs for the total number of gaps (*IP vs IPs*: *b* = −9.16, *p* = 0.009, *d* = −1.23, 95% CI [−1.90, −0.57]), with significantly more gaps produced by the IPs compared to the IP (M = 33.8%, M = 24.7%). For gaps total duration, a main effect of Participant showed that IPs spent more time in gaps than IP (*IP vs IPs*: 0.30, *p* = 0.008, *d* = –1.44, 95% CI [–2.13, –0.76]). A significant interaction with order for ISZ (*b* = 0.43, *p* = 0.021) indicated longer gaps when IPs_ISZ occurred before IP_MAT, though this effect did not survive correction (*p* = 0.11) (Fig. 3).

### Pause

#### Dyad level

**Fig. 4:**
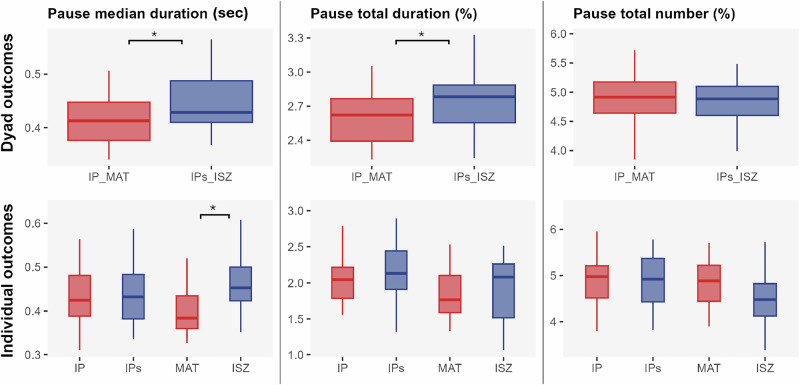
Boxplots of pause metrics at dyadic and individual levels. The dyadic level is illustrated on the top row and the individual level on the bottom row. The first column displays the median duration of pause (in seconds), the second column shows the total pause duration (in %), and the third column represents the total number of pauses (in %). Red boxplots correspond to dyads where the interacting participant was IP and MAT (IP_MAT), while blue boxplots correspond to dyads involving IPs and ISZ (IP_ISZ). Individual-level comparisons are made between IP and IPs and between MAT and ISZ. * denotes *p* < 0.05, **p < 0.01, and ***p < 0.001.

The Linear Mixed Model results indicated a significant effect of the group on pauses median duration (*IP_MAT vs IPs_ISZ*: *b* = 0.042, *p* = 0.049, *d* = –0.76, 95% CI [–1.45, –0.08]), and pauses total duration (*IP_MAT vs IPs_ISZ*: *b* = 0.152, *p* = 0.049, *d* = 0.73, 95% CI [–1.43, –0.05]). Overall, the IPs_ISZ dyads had significantly longer median pauses (M = 0.45 ms, M = 0.26 ms) and a greater total duration of pauses (M = 2.77%, M = 2.61%) compared to the IP_MAT dyad (Fig. 4).

#### Individual level

The Linear Mixed Model results indicated a significant effect of the participant on the pause median duration for ISZ vs MAT (*ISZ vs MAT*: *b* = 0.079, *p* = .04, *d* = 1.13, 95% IC [0.47, 1.78]), with the ISZ producing significantly longer median pauses compared to the MAT participant (M = 0.48%, M = 0.40%) (Fig. 4). A significant order effect showed longer pauses when the interaction started with ISZ (*p* = 0.042), though not significant after correction (*p* = 0.14).

### Correlation with willingness for future interactions

At the end of the interaction, the IP rated their willingness to engage in future interactions with MAT or ISZ. Correlations were conducted on variables that showed significant differences at the dyad level, as these variations indicate meaningful distinctions in interaction dynamics. Both dyadic outcomes (overall conversation synergy) and individual outcomes (partner fluency) were analyzed, as the IP’s willingness may be influenced by the flow of their partner’s speech.

#### IPs and IP willingness (using dyad outcome)

**Fig. 5:**
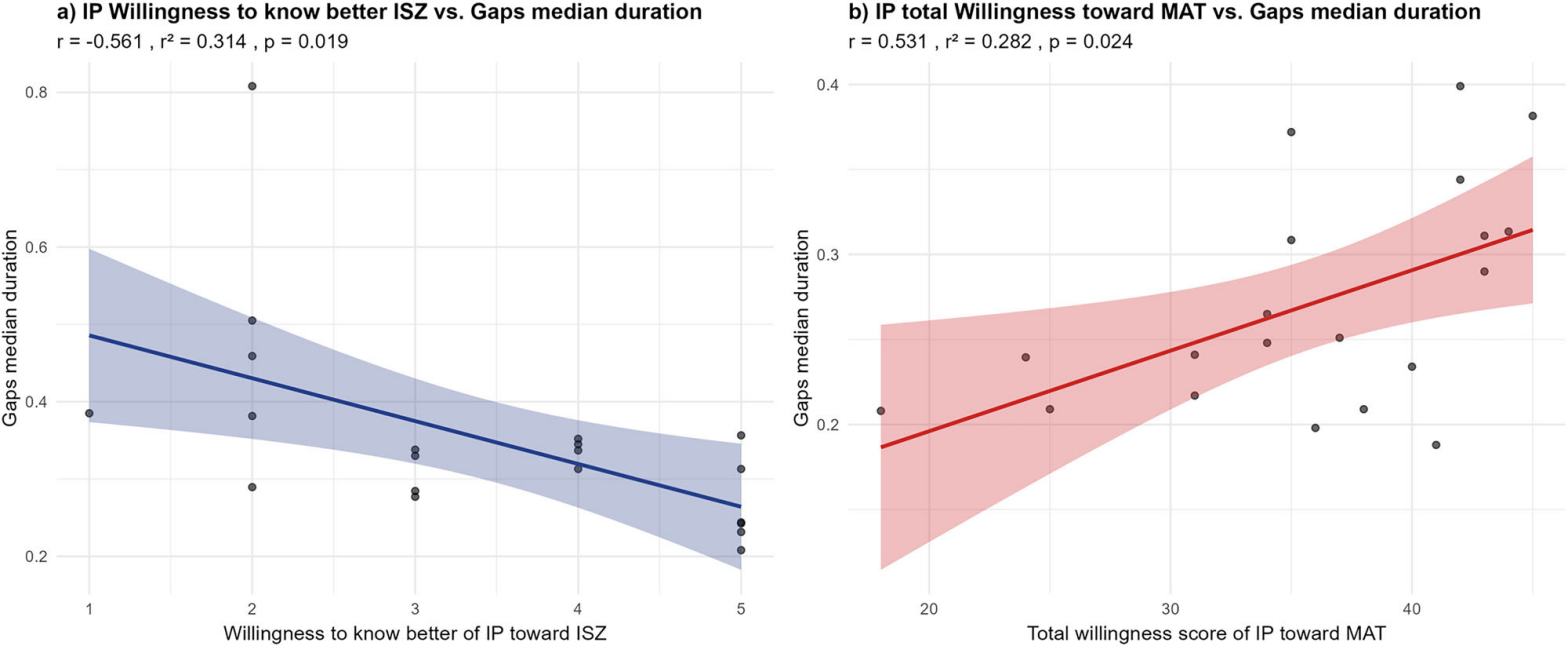
Correlation between willingness to interact and turn-taking outcomes. **a** Rating of IPs toward their ISZ partner; **b** Rating of IPs toward their MAT partner.

Among the 9 items of the willingness questionnaire, we only found one significant correlation between the IPs’ “willingness to know better” and the dyad gaps’ median duration (*p* = 0.019; *R*² = 0.314). While only 31% of the variability of “Willingness to know better” is explained by the duration of gaps, the significant correlation suggests that longer gaps in the conversation negatively affect the IPs’ perception and their will to know better ISZs (Fig. 5a).

Concerning the IP’s willingness to interact with the MAT, we found a significant correlation between the IP’s total willingness and the dyad gaps’ median duration (*p* = 0.024; *R*² = 0.282). Longer gaps seem to be associated with a higher IP’s willingness to engage in future interaction with the MAT (Fig. 5b).

#### IPs and IP willingness (using ISZ and MAT outcome, respectively)

There was no significant correlation between IPs’ willingness and the ISZs’ outcome, as well as between IP’s willingness and the MATs’ outcome.

### Correlation with psychopathological variables (PANSS), medication, and duration of the disease

Due to missing data on three PANSS surveys, correlations were conducted using 17 dyadic outcomes and 17 individual conversation outcomes of ISZ and their interacting partner (IPs) (as the ISZ’s psychopathological symptoms, medication, and duration of the disease may have shaped their speech flow, they could also have impacted their partner’s speech).

#### PANSS scores and dyad outcome

Several significant correlations were found between the IPs_ISZ dyad outcome and the PANSS scores. We especially found that “blunted affect”, “lack of spontaneity and flow of conversation”, and “motor retardation” were associated with a higher number of gaps, longer pause durations, and a lower total number of overlaps. Moreover, the “depression” item was correlated with an increase in gaps in median duration and pause total duration (Table 2) (see Supplementary Fig. 1 for the full correlation heatmap). No correlation was found between turn-taking metrics, medication, and the duration of the disease.

**Table 2:** Table displaying the PANSS items that showed significant correlations with dyads’ outcomes.

PANSS Variable	Gaps median duration	Gaps total duration	Gaps total number	Overlaps the total number	Pause median duration	Pause total duration
Blunted affect	-	-	0.59*	–0.6*	0.72*	0.65*
Lack of flow of conversation	-	-	0.65*	–0.67*	0.64*	-
Depression	0.69*	-	-	-	-	0.72*
Motor retardation	-	-	0.57*	–0.61*	0.66*	0.76*

Values represent Pearson correlation coefficients between PANSS scores and dyadic outcomes. *p* < 0.05 is marked with *. Non-significant correlations are not reported.

#### PANSS scores and individual ISZ, IPs' flow of speech

We found significant links between ISZ’s psychopathological symptoms and speech flow. Blunted affect was associated with fewer overlaps, motor retardation with more gaps and fewer overlaps, and depression with longer gaps, pauses, and fewer overlaps (Table 3). ISZ symptoms also affected their partner’s speech: increased gap duration in the IP’s speech correlated with ISZ’s blunted affect and persecution, while longer pauses in the IP’s speech were linked to ISZ’s motor retardation, lack of spontaneity, and blunted affect (Table 4) (see Supplementary Figs. 2 and 3 for the full correlation heatmap). No correlation was found between turn-taking metrics, medication, and disease duration.

**Table 3:** Table displaying the PANSS items that showed significant correlations with ISZ’s speech flow.

PANSS Variable	Gaps median duration	Gaps total duration	Gaps total number	Overlaps the total number	Pause median duration	Pause total duration
Blunted affect	-	-	-	–0.62*	-	-
Depression	0.6*	0.59*	0.61*	–0.62*	0.59*	-
Motor retardation	-	-	0.62*	–0.66*	-	-

Values represent Pearson correlation coefficients between the PANSS items that showed significant correlations with ISZ’s speech flow. *p* < 0.05 is marked with *. Non-significant correlations are not reported.

**Table 4:** Table displaying the PANSS items that showed significant correlations with IP’s speech flow.

PANSS Variable	Gaps median duration	Gaps total duration	Gaps total number	Overlaps the total number	Pause median duration	Pause total duration
Persecution	0.68*	-	-	-	-	-
Blunted affect	0.62*	-	-	-	0.78*	-
Lack of flow of conversation	-	-	-	-	0.67*	-
Motor retardation	-	-	-	-	0.69*	-

Values represent Pearson correlation coefficients between the PANSS items that showed significant correlations with IP’s speech flow. *p* < 0.05 is marked with *. Non-significant correlations are not reported.

## Discussion

The current study aimed to assess the turn-taking fluency between individuals with schizophrenia (ISZ) and controls and its relationship with social outcomes, psychotic symptomatology. We recruited 20 ISZ, 20 interacting partners (IP), and 20 matched participants (MAT). Blind to the ISZ diagnosis, each IP interacted once with an ISZ and once with a MAT. Consistent with our first hypothesis, we found significant differences between the IP_MAT and the IPs_ISZ dyads in all overlap, gap, and pause dimensions.

Concerning the overlap metric, our results indicated no difference in duration but a significant decrease in the total number of overlaps for the IPs_ISZ dyads compared to the IP_MAT dyads. This finding contradicts our hypothesis and challenges Lucarini et al., who found more overlaps in conversations with individuals with schizophrenia compared to controls^37^. At first glance, their results indicate that the control group minimized simultaneous speech to a greater extent, supporting the rules of Sacks et al.’s turn-taking model in which overlaps indicate a violation of turn-taking norms^15,63^. However, simultaneous talk is widespread in conversations^63^, and certain cooperative overlaps are more reflective of relationship expression dynamics than indicators of disrupted turn-taking^27,63,64^. This is the case of terminal overlaps that occur when a listener begins speaking as the other finishes, either by anticipating turn completion through verbal cues (lexico-syntactic, semantic, content) or by reacting to verbal or nonverbal turn-yielding signals^19,53,65^. Our results highlight that the IPs, rather than the ISZs, showed fewer overlaps, suggesting that IPs may have struggled to detect turn completions, possibly due to a lack of clear verbal and nonverbal cues from their partners. This interpretation aligns with previous studies that found reduced nonverbal behaviors, such as fewer hand gestures^66,67^, decreased rate of head movement^68^, increased flight behavior^69^, and impaired prosodic expression of emotion^70^ in ISZs.

We propose that if ISZs’ partners struggle to detect turn-taking cues, they may delay their response until they are certain the turn is over, leading to longer gaps, consistent with findings that reacting to silence lengthens transitions^56^. It also aligns with our findings of more and longer gaps in conversations with ISZs, especially when they initiated the session. However, though our hypothesis did indeed anticipate longer gaps, we did not expect to find an increased number of gaps, as suggested by the findings of Lucarini et al.^37^. We believe this discrepancy stems from contextual differences. Our study involved free conversation between IPs and ISZs, while Lucarini et al. analyzed semi-structured interviews led by psychiatrists aware of the diagnosis^37^. The higher overlap rates in their study may reflect the psychiatrist’s more directive role, often redirecting the conversation and interrupting the ISZs^71^, thus reducing gaps. This highlights that turn-taking rules are socially constructed and depend on the context and roles of participants^72^. Moreover, we found that only ISZ’s partners significantly increased their gap number compared to their conversations with MAT’s participants. Like Howes and Lavelle^36^, who found longer gaps only in ISZs’ partners, our results show that IPs produced more and longer gaps with ISZs than with MAT. ISZs also tended to produce longer gaps, especially when IPs_ISZ occurred first, suggesting coordination difficulties extend beyond the individual and are amplified when both partners lack task experience.

Differences in fluency in the IPs_ISZ dyads are also confirmed by the longer pauses found in those dyads compared to IP_MAT, aligning with our hypothesis on the pause duration metric. Specifically, we found ISZ participants to drive this dyadic increase, producing significantly longer pauses than MAT participants. Our findings align with previous research on speech fluency in ISZ during monologue tasks^73,74^ as well as studies employing dialog-based tasks, such as interviews^75–77^. However, since the latter often do not distinguish between pauses within speech and pauses between speakers, direct comparisons with our results remain challenging. Nevertheless, we can suggest that the increased pauses in ISZs’ speech may be linked to difficulties in structuring and planning their discourse, retrieving and selecting appropriate words, and organizing their thoughts, particularly at moments requiring sentences or ideas to be connected or transitioned^78,79^.

Overall, we hypothesized that these differences in overlaps, gaps, and pauses should be associated with poorer social outcomes in the IPs_ISZ dyad, such as a reduced willingness for future interaction. Our results indicate that only IPs’ willingness to know their ISZ partners’ items correlated with gap duration: as gap length increases, their interest in knowing their partner declined, consistent with research showing reduced connectedness in conversations with longer gaps^59^. In contrast, longer gaps in IP_MAT dyads were linked to a greater willingness to engage in future interactions, an unexpected finding. As Templeton et al. found in friends’ conversations, such pauses may support reflection. With ISZs, gaps might feel more awkward, similar to those in conversations between strangers^59^. This discrepancy suggests that the social impact of conversational gaps depends on the speaker and context; some perceive pauses as awkward, others as thoughtful. Additionally, the meaning attributed to these latencies can be shaped by contextual factors such as the topic of discussion, the nature of the relationship between participants, social norms, and the content exchanged^80^. These elements may also influence how willing a person is to continue the interaction. Moreover, it is worth noting that these correlations were observed using dyadic outcomes, emphasizing that individual participants may base their decision to continue the interaction on their overall perception of the conversation’s synergy, rather than solely on their partner’s speech flow.

Finally, our last hypothesis predicted that the differences in overlaps, gaps, and pauses observed in IPs_ISZ would be associated with the psychological dimensions of ISZ symptomatology. As expected, ISZ’s psychopathological symptoms affected their speech flow and were correlated with gaps and pauses in their partner’s dialog, influencing both participants and the overall conversation outcome. We found that higher numbers of gaps, longer pauses, and fewer overlaps were linked to negative symptoms of schizophrenia, such as blunted affect, motor retardation, and depression. These results support our hypothesis of reduced speech flow in ISZs, likely due to diminished verbal and nonverbal cues. Significant correlations with blunted affect and motor retardation on the PANSS scale, marked by reduced emotional responsiveness and slower motor activity and speech, further align with prior research linking negative symptoms to turn-taking patterns^37,81^.

While our results provide new insight into the flow of turn-taking in conversations with individuals diagnosed with schizophrenia, there are also limitations to consider. First, the small number of dyads may limit the generalizability of our findings, as it can increase data variability and reduce statistical power. This is particularly important when interpreting correlations between symptoms and conversational dynamics in the schizophrenia group, as reduced power may obscure meaningful associations. Second, French ethical regulations prevented collecting racial or ethnic data, limiting interpretation of willingness to interact, since cultural or dialectal factors may have influenced responses. Third, assigning a different partner to each dyad likely added individual variance, influencing dynamics and limiting generalizability. Fourth, the first four dyads used lapel microphones, while the rest used headsets. This inconsistency, along with lower lapel sound quality, may have introduced variability in participant behavior. Fifth, participants’ education levels were not recorded, despite potential effects on conversational behavior. Furthermore, although MoCA and BDI-II scores were obtained, the absence of medical comorbidity data limits full interpretation of variability in communication. Sixth, we found no correlation between disorganized symptoms, likely due to the use of a non-specific tool (sub-scale disorganization of the PANSS^82^). Future studies should examine disorganization/turn-taking relationships using specific tools such as the Thought, Language, and Communication scale^83^ or the Thought and Language Disorder scale^84^. Seventh, although we tried to control the content of the interactions by directing participants to discuss topics they both enjoy, some participants may have encountered more difficulty finding common ground and topics of conversation that motivated them. The content of the conversation was not analyzed in this study, although it could have played a role in the form and dynamics of turn-taking^28^. Eighth, turn-taking is multimodal and is shaped by both verbal and nonverbal cues^85^. Therefore, while this study was motivated by looking at the flow of the conversation only through specific conversational patterns (overlaps, gaps, pauses), other verbal and nonverbal signals could have shaped our findings. Future research should explore whether the reduced overlap proportion and increased gap and pause duration found in the IPs_ISZ dyads could be linked to lower gesture frequency in ISZ participants.

## Conclusion

To conclude, our study supports the idea that turn-taking fluency is impaired in free dialog involving individuals diagnosed with schizophrenia because of an increased number and length of gaps and pauses. On the individual level, some outcomes, like overlap number, were mainly driven by one participant, highlighting the value of analyzing both individual and dyadic levels. Moreover, the overall conversational flow at the dyadic level influenced participants’ willingness to engage in future interactions. These dyadic outcomes were also shaped by ISZ symptoms, highlighting their impact on conversational quality. These insights offer new directions to improve therapeutic communication with individuals diagnosed with schizophrenia, including turn-taking techniques like signaling when it’s their turn to speak, pausing to give them time to respond, and prompting them to take their turn, all to support social engagement.

## Supplementary information

Supplementary information

## Data Availability

The annotation data supporting the findings of this study are available in the Open Science Framework at https://osf.io/g97hn/, while the original audio recordings are available upon request from the corresponding author, T.F. The audio data are not publicly available as they may compromise participant consent and confidentiality.

## Code availability

The code used to extract turn-taking metrics from the audio and to perform statistical analyses is available on the Open Science Framework at https://osf.io/g97hn/.
