# Supplementary material for: Turn-taking fluency in free conversations with individuals diagnosed with schizophrenia
Source: Schizophrenia (Heidelb). 2025 Nov 4;11(1):130. doi: 10.1038/s41537-025-00678-y (PMC12586683; doi:10.1038/s41537-025-00678-y)
Supplement: Supplementary file 1 — Supplementary information [file 41537_2025_678_MOESM1_ESM.docx]

**Supplementary Material –**

**Table 1**: Significant model comparisons to test for dyad and order role

| Metric | Model_1 | Model_2 | AIC | BIC | LogLik | Chi2 | p_value |
| --- | --- | --- | --- | --- | --- | --- | --- |
| Gaps_median  _duration | null_model | dyad_model | -91.126 | -84.371 | 49.563 | 6.186 | 0.013 |
| Gaps_median  _duration | dyad_order_model | interaction_model | -96.851 | -86.718 | 54.425 | 8.818 | 0.003 |
| Gaps_tota  l_duration | null_model | dyad_model | 57.884 | 64.639 | -24.942 | 11.295 | 0.001 |
| Gaps_total  _duration | dyad_order_model | interaction_model | 53.414 | 63.547 | -20.707 | 7.803 | 0.005 |
| Gaps_total  _number | null_model | dyad_model | 326.619 | 333.375 | -159.310 | 7.017 | 0.008 |
| Overlaps_total  _number | null_model | dyad_model | 325.969 | 332.724 | -158.984 | 4.700 | 0.030 |
| Pause_median  _duration | null_model | dyad_model | -111.854 | -105.098 | 59.927 | 5.570 | 0.018 |
| Pause_total  _duration | null_model | dyad_model | 6.927 | 13.683 | 0.536 | 5.047 | 0.025 |

*Note:* This table presents the significant results of likelihood ratio tests used for model selection. For each interaction metric, five nested linear mixed-effects models were compared: a null model (random intercept for triad only), a model with order, a model with Dyad, an additive model with both predictors, and a model including the Dyad × order interaction. These tests were used to guide model selection only.

**Table 2**: Significant model comparisons to test for participant and order role

| **Metric** | **Model_1** | **Model_2** | **AIC** | **BIC** | **LogLik** | **Chi2** | **p_value** |
| --- | --- | --- | --- | --- | --- | --- | --- |
| Overlaps_total  _number | null_model | Part_model | 570.318 | 584.610 | -279.159 | 11.867 | 0.008 |
| Gaps_median  _duration | Part_order_model | interaction_model | -148.738 | -124.918 | 84.369 | 9.272 | 0.026 |
| Gaps_total  _number | null_model | Part_model | 570.908 | 585.200 | -279.454 | 14.636 | 0.002 |
| Gaps_total  _duration | null_model | Part_model | 82.265 | 96.557 | -35.132 | 24.769 | 0.000 |
| Gaps_total  _duration | Part_order_model | interaction_model | 73.903 | 97.723 | -26.951 | 15.680 | 0.001 |
| Pause_median  _duration | null_model | order_model | -177.067 | -167.539 | 92.534 | 4.723 | 0.030 |
| Pause_median  _duration | null_model | Part_model | -180.804 | -166.512 | 96.402 | 12.459 | 0.006 |
| Pause_median  _duration | Part_model | Part_order_model | -183.542 | -166.868 | 98.771 | 4.738 | 0.029 |
| Pause_total  _number | null_model | Part_model | 147.145 | 161.438 | -67.573 | 9.693 | 0.021 |
| Pause_total  _duration | null_model | Part_model | 89.779 | 104.071 | -38.890 | 9.119 | 0.028 |

*Note*: This table presents the significant results of likelihood ratio tests used for model selection. For each interaction metric, five nested linear mixed-effects models were compared: a null model (random intercept for triad only), a model with order, a model with Participant, an additive model with both predictors, and a model including the Participant × order interaction. These tests were used to guide model selection only.


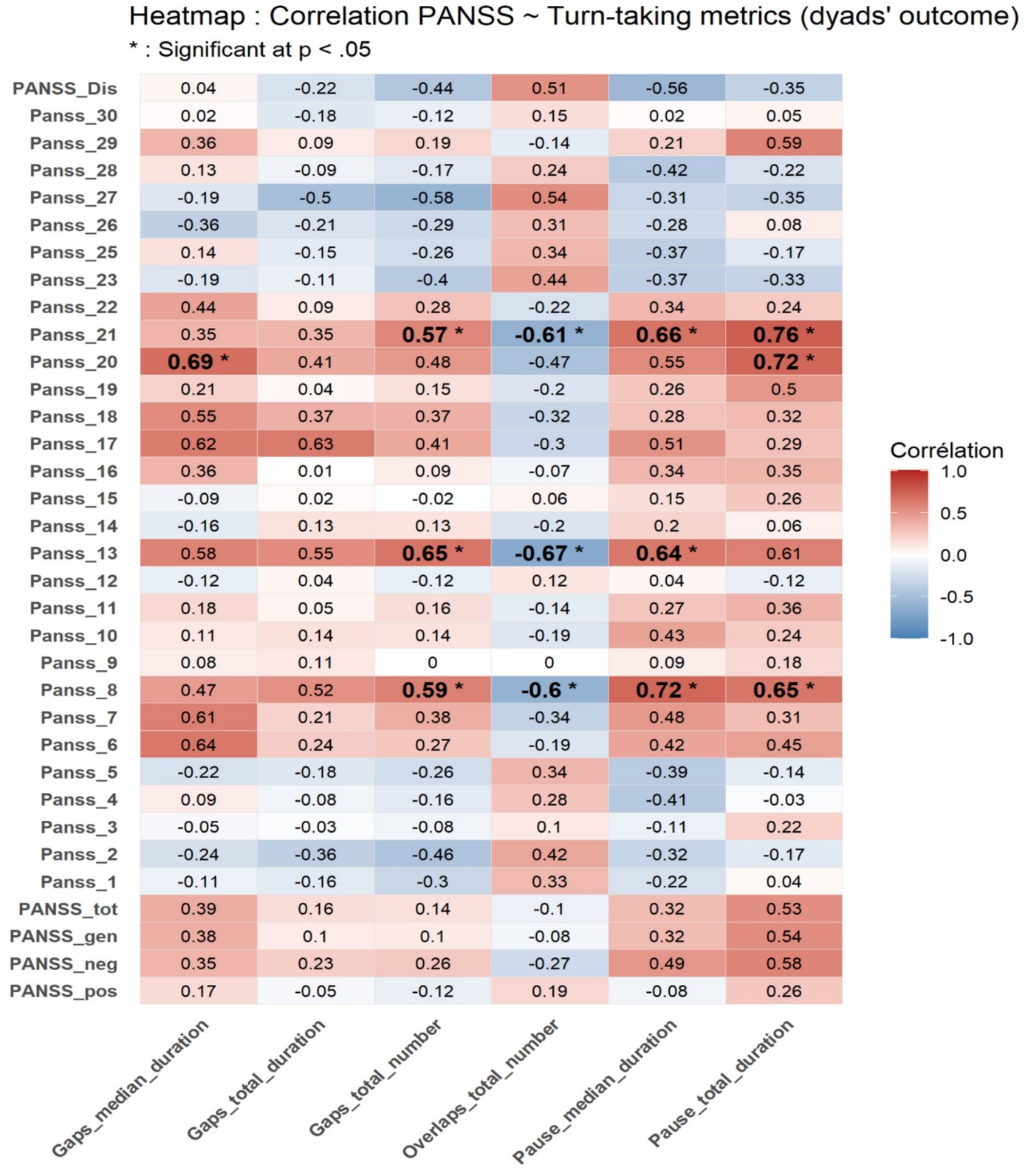


**Figure 1:** Heatmap of correlations between PANSS items/subscales and turn-taking metrics (dyads’ outcome) in the IPs_ISZ group.
The color scale indicates Pearson correlation coefficients (red = positive, blue = negative). Asterisks (*) mark significant correlations at p < .05. PANSS subscales correspond to PANSS total, general, negative, positive, and disorganization.


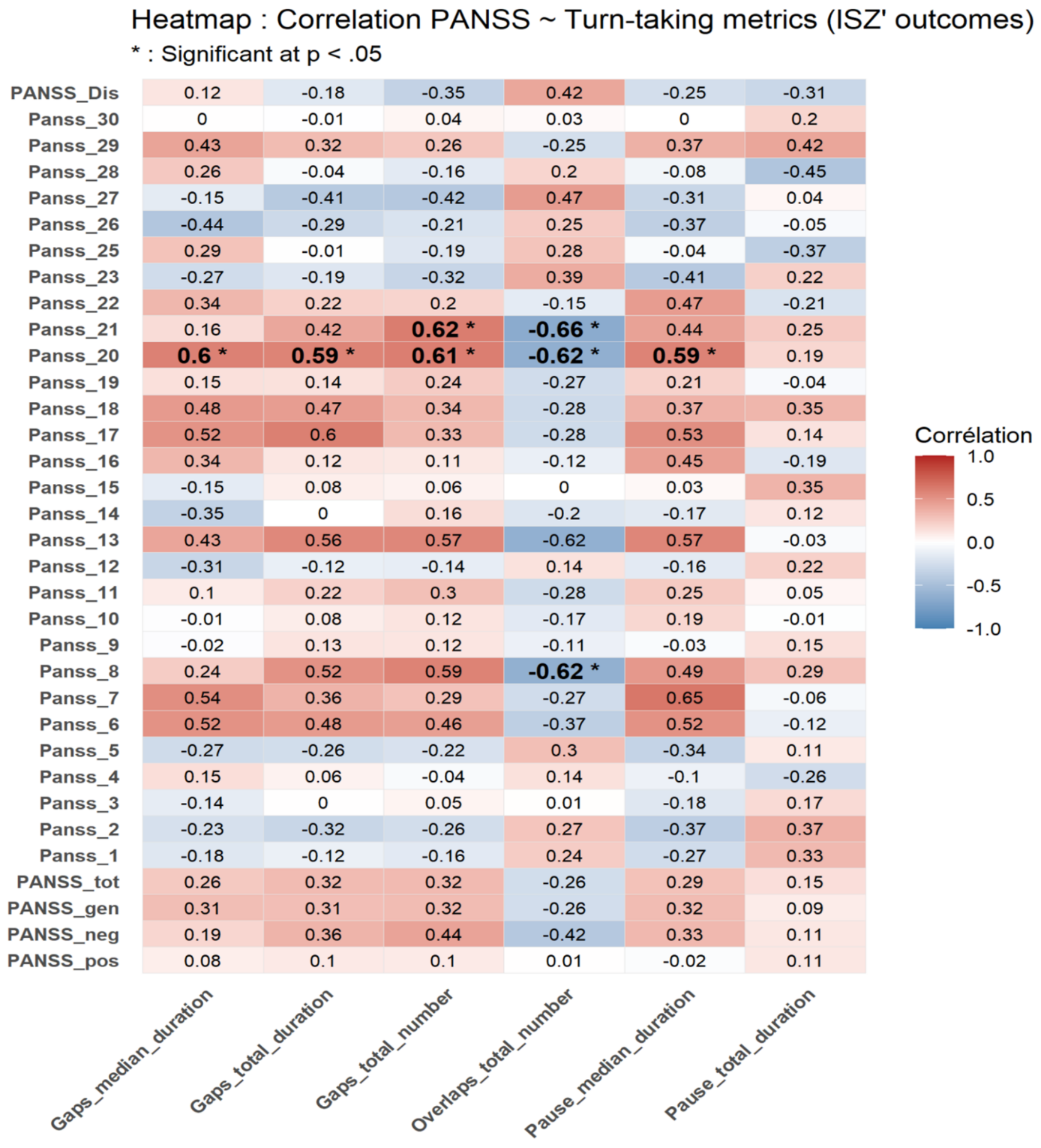


**Figure 2:** Heatmap of correlations between PANSS items/subscales and turn-taking metrics (ISZ’ outcome) in the IPs_ISZ group.
The color scale indicates Pearson correlation coefficients (red = positive, blue = negative). Asterisks (*) mark significant correlations at p < .05. PANSS subscales correspond to PANSS total, general, negative, positive, and disorganization.


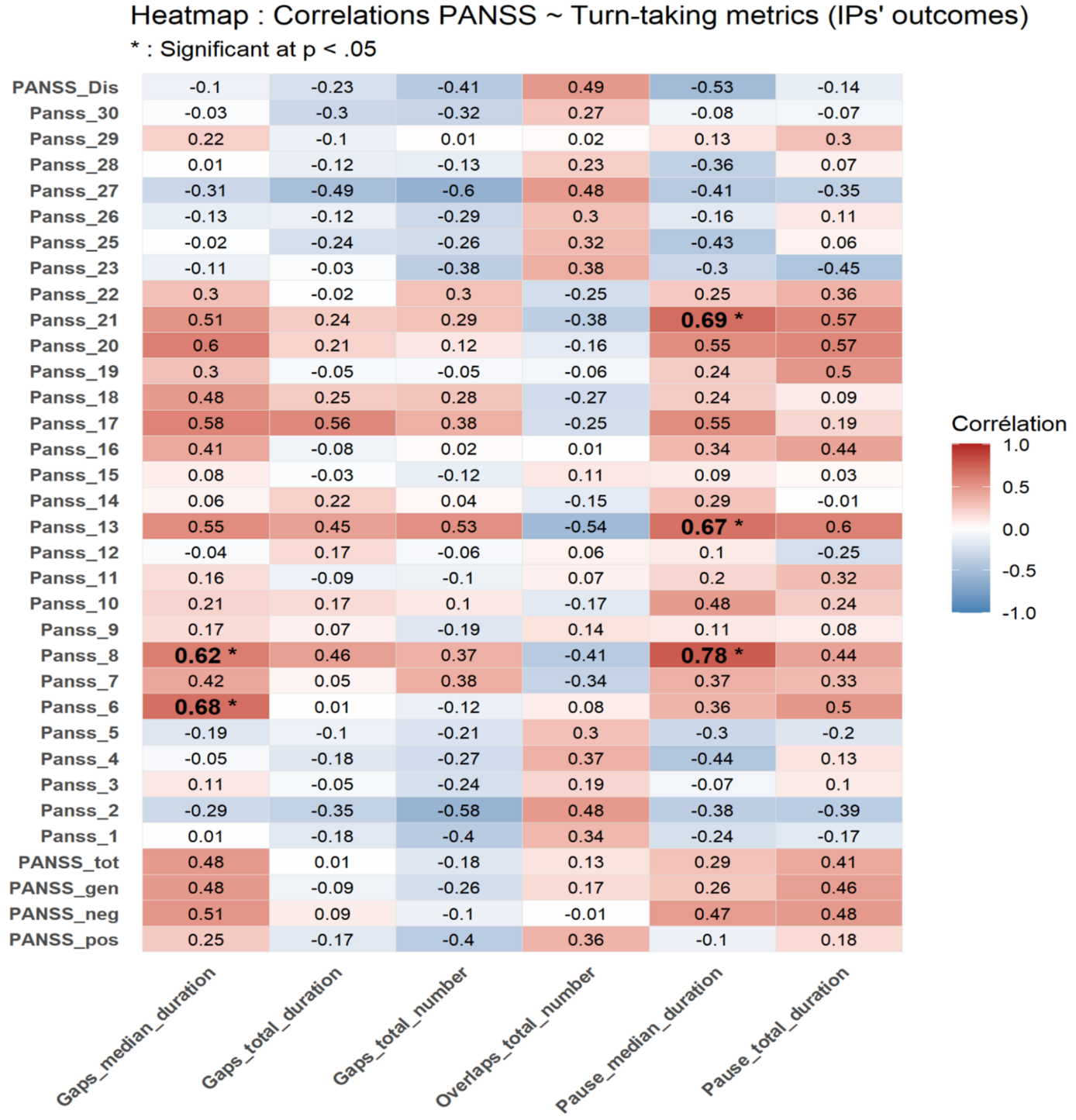


**Figure 3:** Heatmap of correlations between PANSS items/subscales and turn-taking metrics (IPs’ outcome) in the IPs_ISZ group.
The color scale indicates Pearson correlation coefficients (red = positive, blue = negative). Asterisks (*) mark significant correlations at p < .05. PANSS subscales correspond to PANSS total, general, negative, positive, and disorganization.
